# Electroporation on microchips: the harmful effects of pH changes and scaling down

**DOI:** 10.1038/srep17817

**Published:** 2015-12-14

**Authors:** Yang Li, Mengxi Wu, Deyao Zhao, Zewen Wei, Wenfeng Zhong, Xiaoxia Wang, Zicai Liang, Zhihong Li

**Affiliations:** 1 Institute of Molecular Medicine, Peking University, Beijing 100871, China; 2 National Key Laboratory of Science and Technology on Micro/Nano Fabrication, Institute of Microelectronics, Peking University, Beijing 100871, China; 3 Department of Engineering Science and Mechanics, The Pennsylvania State University, State College, PA 16801, USA; 4 National Center for Nanoscience and Technology, Beijing 100190, China

## Abstract

Electroporation has been widely used in delivering foreign biomolecules into cells, but there is still much room for improvement, such as cell viability and integrity. In this manuscript, we investigate the distribution and the toxicity of pH changes during electroporation, which significantly decreases cell viability. A localized pH gradient forms between anode and cathode leading to a localized distribution of cell death near the electrodes, especially cathodes. The toxicity of hydroxyl ions is severe and acute due to their effect in the decomposition of phospholipid bilayer membrane. On the other hand, the electric field used for electroporation aggravates the toxicity of hydroxyl because the electropermeabilization of cell membrane makes bilayer structure more loosen and vulnerable. We also investigate the side effects during scaling down the size of electrodes in electroporation microchips. Higher percentage of cells is damaged when the size of electrodes is smaller. At last, we propose an effective strategy to constrain the change of pH by modifying the composition of electroporation buffer. The modified buffer decreases the changes of pH, thus enables high cell viability even when the electric pulse duration exceeds several milliseconds. This ability has potential advantage in some applications that require long-time electric pulse stimulation.

Electroporation has been a powerful method for the delivery of a large variety of foreign biomolecules into target cells^1^,^2^. This method has shown its capacity in introducing DNA plasmids or other vectors in various types of applications, ranging from cells to yeast and E-coli (such as ref. 3, 4, 5, 6, 7, 8, 9, 10), from *in vitro* studies to *in vivo* tissues^11^,^12^,^13^,^14^,^15^,^16^,^17^. Because of the advantages of broad applicability, rapidity, technical simplicity and avoidance of using toxic chemicals, electroporation is becoming a promising tool in the research of gene therapy and DNA vaccination. Most recently, researchers are using electroporation to generate human induced pluripotent stem cells (iPSCs)^18^,^19^,^20^.

At the early stage, the cuvette type electroporation device was proposed and commercialized by several companies, such as Eppendorf (Eppendorf, Hamburg, Germany) and Bio-Rad (Bio-Rad Laboratories Inc., Hercules, California, U.S.A.). Later some novel instruments were developed, such as the capillary based electroporation device proposed by Kim *et al.*^21^, which was implanted to a commercial instrument, Neon^®^ Transfection System by Invitrogen (Thermo Fisher Scientific Corporation, Massachusetts, USA). Microfabrication technology was utilized in developing electroporation devices^22^,^23^. He *et al.*^24^,^25^ proposed an electroporation chip that contained micro electrodes by patterning gold on the glass substrates. The rectangle comb-formatted electrodes enabled small gaps between anodes and cathodes, thus reduced the voltage. García-Sánchez *et al.*^26^ used round-shaped comb electrodes, which could be inserted into the wells on the multi-well plate. Recently, Xu *et al.*^27^ used the comb electrodes on a printed circuit board and assembled a novel multi-well plate. Huang *et al.*^28^ proposed a novel pattern of microelectrodes. The microchip with annular interdigitated electrodes also showed ability to obtain high transfection efficiency as well as to integrate with multi-well plate. Recently, more and more microscale devices have been proposed (such as ref. 29, 30, 31), in which the gaps of electrodes can be as smaller as tens of micrometers. Furthermore, nanoscale devices have been used for electroporation by scaling down the size of electrodes^32^,^33^,^34^ or using nano structures such as nanowires^35^,^36^,^37^,^38^.

However, in all of these devices, a dramatic pH change is always accompanying with electroporation because of an electrolytic nature. The pH value of solution should be close to intracellular pH in order to get optimal transfection results^39^,^40^. It is well known that the changes of pH during electroporation are harmful to cells, and some primary researches have revealed the changes of pH in the solution, mainly located near the electrodes. Friedrich *et al.*^41^ presented the pH-gradient produced by electrolysis of water during electroporation through aluminum electrodes in the cuvette type devices. Kim *et al.*^21^ studied the changes of pH in cuvette and capillary based electroporation device. Localized pH changes were observed. And cells near the electrodes were dramatically damaged, swollen, and eventually ruptured. They claimed that the high level of electric pulse might accelerate this phenomenon, but did not show convincing results to support that. Huang *et al.*^28^ monitored the changes of pH on annular interdigitated microchip during electroporation. Furthermore, they found that dead cells were seen to cluster around the cathodes. Turjanski *et al.*^42^ and Maglietti *et al.*^43^ presented the experimental results and theoretical predictions of hydrogen and hydroxyl ions transport in agar gels and tissue during electroporation. A dramatic pH gradient near the electrodes was testified.

On the other hand, researchers show great interests in how to decrease the damage of pH changes during electroporation. Meir *et al.*^44^ proposed an approach to keep cells away from the effects of electrolysis by using Kapton tape to isolate electrodes. Wu *et al.*^45^ proposed an novel distributed electrode network to neutralize ions by using a tri-phase pulse. Assisting with tri-phase stimulation mode, they found the distributed electrode network was able to avoid the dramatic changes of pH during electroporation.

In this paper, we investigated the distribution of pH changes during electroporation on microchips. We monitored the distribution of cell death caused by pH changes. Localized distribution of cell death was observed near the electrodes. The damage of hydroxyl ions was found to be more dramatic and fatal because the saponification of lipid led to the breakdown and lysis of membrane structure. Furthermore, we explored the application of electric filed and verified that the electric field increased the harmful effects of pH changes. The damage of hydroxyl ions when a 1
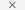
10^5^ V/m electric field existed was significantly more severe than that without electric field. In addition, we investigated the side effects induced by scaling down of microelectrodes. Besides the geometric considerations (since as the size of device decreases, the effective electroporation volume reduces^46^), pH changes was found to become more severe and influent more percentage of electroporation volume as the device size being decreased.

At last, we proposed a modified buffer recipe for electroporation to avoid the side effects of pH changes. The modified buffer contained enough volume of pH buffer agent, which was able to neutralize the booming of hydrogen or hydroxyl ions. With the help of modified buffer, the distribution of cell death was observed to decrease, and the viability of electroporation maintained as a high level. The modified buffer kept a mild pH value even when the electric stimulation was as long as 5 ms. This capability can be beneficial to some applications which require long duration pulse stimulation.

## Results And Discussions

### Localized pH variation

For the application of electroporation, a high electric potential is applied between anode and cathode. Thus the charge and discharge of electrons induces electrolysis of water. The reaction can be described as the following equations.


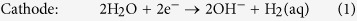



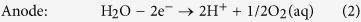



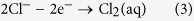



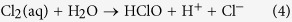


Therefore, a hydrogen/hydroxyl gradient, which can be described as pH gradient, will form transiently between anodes and cathodes. The localized alkaline zone distributes near to cathodes, while the acid zone near to anodes.

**Figure 1:**
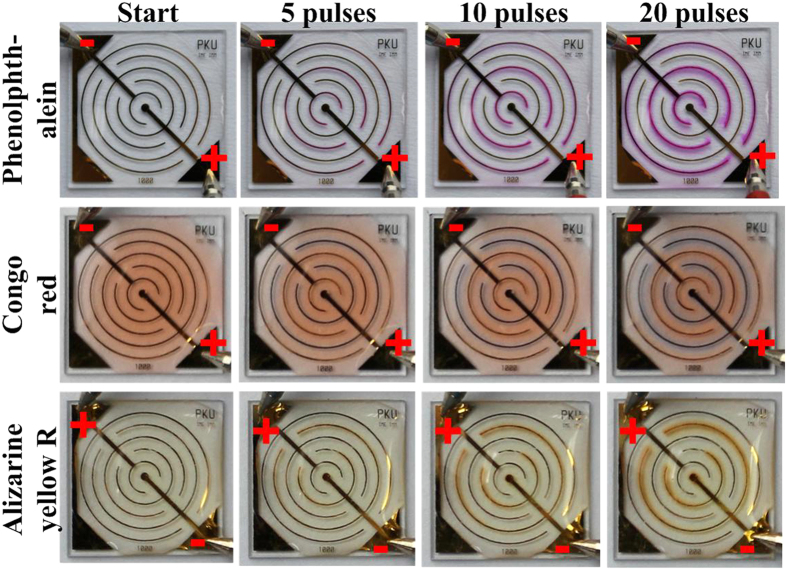
The localized changes of pH near to electrodes during electroporation. Three types of pH indicators manifest localized dramatic acidification or alkalization near to anodes or cathodes. Anode and cathode is indicated by “ + ” or “−” respectively. And the polarity is altered during the experiments to test repeatability.

In order to figure out the distribution of pH changes, we performed electroporation on annular interdigitated microchips using pH indicators. The applied electric field is about 1.0
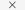
10^5^ V/m, equivalent to the optimum condition proposed by our previous studies^28^,^47^. The results are shown in Fig. 1. As indicated by phenolphthalein (below pH 8.2: colorless, above pH 10: fuchsia) and Congo red (below pH 3.0: blue, above pH 5.2: red), the pH of medium near to cathodes and anodes changes dramatically. As electric stimulation increases, hydroxyl and hydrogen ions accumulate near cathodes and anodes, and diffuse to nearby areas. The change of color (from colorless to fuchsia or from red to blue) indicates that the pH values within cathode and anode areas are at least above 10.0 and below 3.0, respectively.

As a multiple evidence, Alizarine yellow R (below pH 10.1: yellow, above pH 12.0: red) was used, and, furthermore, the polarity of cathode and anode electrodes was altered. The results are also presented in Fig. 1. The color of medium near to cathodes changes correspondingly. That means pH value near cathodes is not only above 10.0, but may even higher than the level of 12.0.

### Cytotoxicity of base and acid

The localized pH changes during electroporation are transient due to the diffusion and migration of hydrogen or hydroxyl ions. Furthermore, in some electroporation protocols, the medium is refreshed after electroporation, thus the influence of pH changes to cells is terminated. However, the transient pH shock is still fatal to cells, especially strong alkaline stimulation. In order to demonstrate the damage of transient pH changes to cells, we treated cells with pH shock.

**Figure 2:**
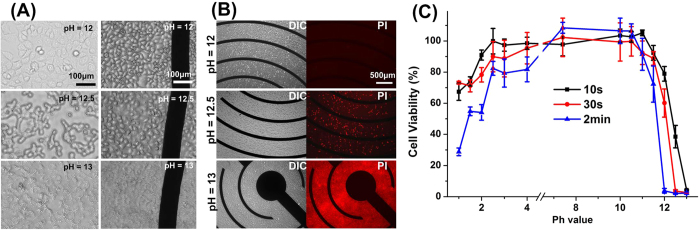
The damage of hydroxyl and hydrogen ions to cells. (**A**) Morphology of HEK-293A (left column) and Hela (right column) cells after pH shock. Most cells exhibit no abnormal morphology when pH = 12. Cells shrink when treated pH is 12.5, with a mild mortality of cells. Lysis of cell happens when pH is 13. (**B**) Differential interference contrast (DIC) and polyimide (PI) fluorescent images of HEK-293A cells after pH shock. (**C**) MTT assay of Hela cells indicates the fatal dose of hydroxyl for cells is pH > 12. Mass mortality of cells happens when pH < 2. All data is obtained from at least three experiments and presented as mean ± standard error.

Figure 2(A) shows the morphology of HEK-293A and Hela cells after treated with alkaline solution for 10 seconds. When the pH value is 12 for alkaline stimulation, both types of cells exhibit no abnormal morphology. For pH value is 12.5, cells shrink obviously after alkaline stimulation. Alkaline stimulation in solution with pH of 13 is fatal and devastating, even for only 10 seconds. Cell lysis happens immediately, with only cell debris remaining on the chip. The PI staining (as shown in Fig. 2(B)) also shows cell lysis happens when pH is 13. Nucleic acids are released in the solution and exposed to PI staining. Fluorescence is observed almost everywhere. The devastating damage of strong base is because phospholipids, which form cell membrane and nucleic membrane, are broke down by hydroxyl ions. Base hydrolysis causes the saponification reaction. Thus decomposition of membrane aggravates mortality of cells.

In addition, cell viability was evaluated by MTT assay, in which the ratio reflects cell viability. The results (Fig. 2(C)) show both acid and base influence cell viability. It can be seen that the longer duration of pH shock, the lower viability of cells. Furthermore, the alkaline environment performs more severe cytotoxicity than the acidic one. When the duration is 2 minutes, more than 50% cells survive at pH of 2 while almost all cells die when pH equals to 12. Interestingly, there is a critical pH value of cell viability. When the pH value is larger than 12, the cell viability decreases steeply. Almost no cell survives when pH value reaches to 13 in all three different pH shock duration experiments.

### Cell death distribution during electroporation

**Figure 3:**
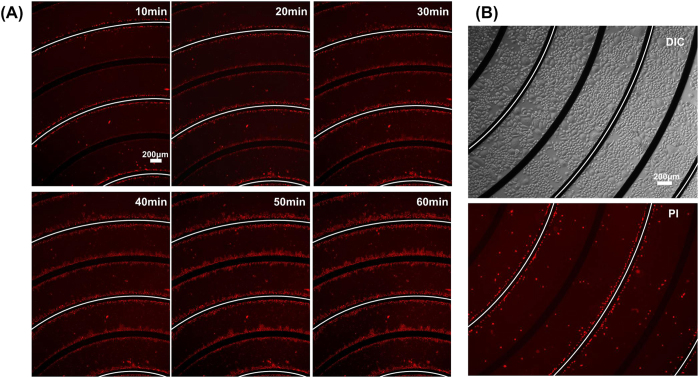
Localized distribution of Hela cell death near to cathode and anode electrodes. (**A**) PI fluorescent images of cells at different time point after electroporation indicate the death of cells. (**B**) Images of cells 24 h after electroporation. The electrodes figured by white line are cathodes, whereas others are anodes.

Then we further investigated the death of cells after electroporation on a microchip with annular interdigitated electrodes by using PI staining. The results are shown in Fig. 3. From 10 minutes after electroporation to one hour after electroporation, PI fluorescence is performed near both cathodes and anodes. However, fluorescence emerges later near anodes than near cathodes (figured with white line) in the beginning, followed by no obvious difference one hour after electroporation (Fig. 3(A)). When observed one hour after electroporation, cells near both anode and cathode shrink and are stained by PI. The width of cell death distribution area is about 100 μm (shown in Supplementary Figure 1). On the other hand, when observed 24 hours after electroporation, PI fluorescence performed near cathodes only, but not near anodes (Fig. 3(B)).

The results indicate that the influence of acidic and alkaline environment to cell viability is different during electroporation. The damage of cells caused by the acidic environment is less deadly and less acute than the alkaline one. Some of the survival cells recover and replace dead cells after 24 hours. However, PI staining is still observed near the cathode electrodes. The distribution of cell death after 24 hours manifests that the damage of base to cells is more severe than that caused by acid. Kim *et al.*^21^ performed a similar research. They treated HeLa cells with the acidic (pH 3) and alkaline R buffer (pH 11) for 5 minutes. They found these cells were severely damaged by the pH change. In our research, cells are exposed to pH change less than 2 minutes, which is more close to the real situation of cell electroporation. Cell viability do not lower too much at pH 3 and pH 11. We further observed during the time course until to 24 hours after electroporation. Moreover, we reveal the damage of cells near anode is less deadly and less acute than near cathode.

### Electric field aggregates OH^−^ toxicity

We found the joint harmful effects of electric field and alkaline environment caused more damage to cells. The electric field applied to cells makes bilayer lipid structure more permeable and loose, thus more vulnerable to be deformed by OH^−^. In order to testify the effects of electric field and further investigate the critical value of electric field intensity, we used another type of microchip to perform electroporation. Four-leaf shaped microchip, which was proposed by M. Wu *et al.*^48^, consists of four hyperbolic shaped electrodes in respective quadrants. When the opposite electrodes are connected together, an annular distribution of electric field is generated (shown in Supplementary Figure 2). The electric field distribution is calculated by COMSOL Multiphysics software. At the central point of microchip, the electric field intensity is zero. The electric field intensity increases linearly as the radius of annular ring increases.

**Figure 4:**
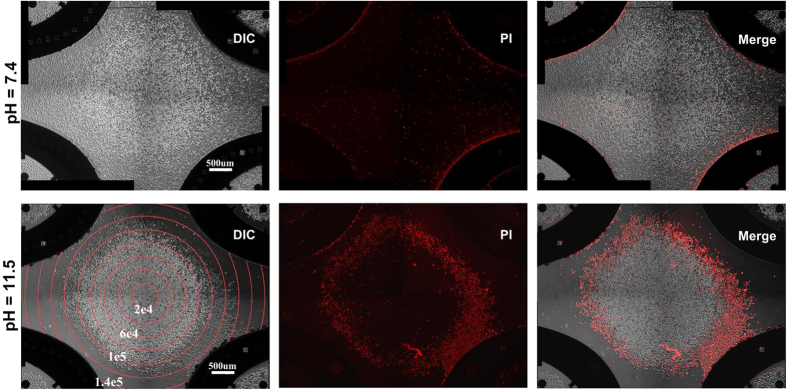
Demonstration of electric field assisted OH^−^ toxicity effects by using four-leaf microchip. Images of HEK-293A cells 1 h after electroporation. Cells are seriously damaged when high electric field intensity and OH^−^ present simultaneously. The red lines represent electric field intensity contour (unit: V/m). The electric field intensity is calculated by COMSOL Multiphysics.

The results are shown in Fig. 4. As a control, the results under pH = 7.4 indicate that cells are slightly damaged by electric field. Only the cells located near to the electrodes are stained by PI. While under the same electric stimulation, an annular cell death region is observed when the pH of buffer is 11.5. As indicated by Fig. 2, pH = 11.5 is not fatal to cells. However, when cells are exposed to a certain electric field intensity, pH = 11.5 will damage the cells. According to the PI fluorescent images, cells are almost not stained in the area where the electric field intensity is below 8
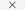
10^4^ V/m. The critical electric field intensity is about 8
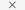
10^4^ V/m ~ 9
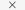
10^4^ V/m. In those areas where the electric field intensity is higher than 1
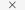
10^5^ V/m, the decomposition of lipid membrane leads to the lysis of cells. Thus when observed one hour after electroporation, most of cells have been disintegrated. The cell debris is removed by replacing the culture medium. Therefore, when an electric field exists (the intensity of electric field is ~ 1
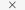
10^5^ V/m), the fatal pH value is 11.5; whereas without electric field cells are damaged when pH value is above 12.5. In other words, the toxicity of hydroxyl ions is increased by 10 times with the aggravation of electric field stimulation.

### Scaling down effects of microchips

In the past few decades, microfabrication technique has facilitated the development of electroporation microchips. The gap between two electrodes can be as small as “cell size”. To decrease the gap between electrodes is beneficial to decrease voltage needed for electroporation. However, the side effects of the scaling down need to be considered. Novickij *et al.*^46^ analyzed the electric field distribution of microchips with varied gap ranging from 50 μm to 500 μm. They found that a wider gap between the electrodes was advantageous for higher electric field homogeneity. Besides the drawback of less homogeneous electric filed, the scaling down of electrodes also contributes to more percentage of pH change segments. Lower transfection efficiency and cell viability is caused by the side effects of electric field non-uniformity and severe pH change toxicity.

**Figure 5:**
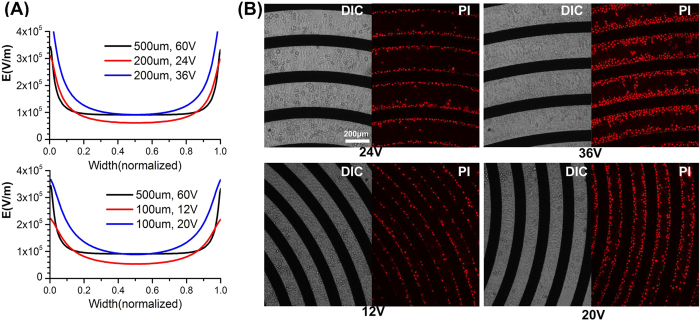
Distribution of electric field and Hela cell death on microchips. (**A**) The distribution curve of electric field intensity at the height 6 μm above the substrate. The width between two electrodes is 500 μm, 200 μm and 100 μm respectively. (**B**) Hela cell death distribution on microchips. The distributions of dead cells expand due to larger percentage of strong and non-uniform electric field. The upper part is 200 μm width and the lower part is 100 μm width.

When the gap between electrodes is 200 μm or 100 μm, the electric field is much less uniform than that of 500 μm microchips (the detailed simulation is shown in Supplementary Figure 3). Figure 5(A) presents the distribution of electric field intensity along the line 6 μm above the substrate. The x axis represents normalized width between two adjacent electrodes. For 500 μm electrodes, the optimized voltage is about 60 V. About 80% of the area is exposed to a uniform electric field. The proper electric field intensity is about 1
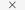
10^5^ V/m. For 200 μm electrodes, the percentage of uniform electric field is much less. Another issue is the intensity of electric field. According to the calculation of scaling down, the corresponding voltage for 200 μm electrodes should be 24 V (as for 500 μm electrodes the voltage is 60 V). However, when 24 V is applied, the electric field intensity decreases to 6
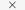
10^4^ V/m, which is not enough for electroporation. That means the electric field intensity is not the optimal condition. Thus the transfection efficiency may not be acceptable. In order to generate enough electric field intensity, the voltage needs to be modified to 36 V. However, the increase of voltage leads to higher electric field intensity near the electrodes. On the other hand, the percentage of area with proper electric field intensity is less than 50%. For 100 μm electrodes, the situation is even worse. The modified voltage is 20 V, and the percentage of proper electric field intensity is less than 40%.

Due to a higher percentage of non-uniform electric field distribution, the side effects caused by pH changes are more severe when using microchips with smaller width between electrodes. Figure 5(B) shows the results of 200 μm and 100 μm microchips. The images are acquired one hour after electroporation. In general, when the modified voltage is applied to microchips (36 V for 200 μm electrodes, 20 V for 100 μm electrodes), higher percentage of cells are damaged. The distribution of PI stained cells occupies nearly 40% of the electrodes aperture. At this case, cell viability will decrease. Thus the electroporation efficiency will also be undermined. Besides, when observed 24 h after electroporation, the phenomenon is similar to that on 500 μm microchips. PI fluorescence is observed mostly near cathodes (shown in Supplementary Figure 4). The results testify the conclusions mentioned before.

### Strategy to constrain pH changes

Electric field and pH changes induced by electrolysis near electrodes co-contribute to cell death during cell electroporation. Here we proposed a strategy to constrain the changes of pH and thus improve cell viability during cell electroporation by using a modified electroporation buffer.

In many applications, phosphate buffered saline is used to keep the pH of solution less vulnerable, known as PBS buffer. For example, the Eppendorf hypoosmolar electroporation buffer contains phosphate buffered saline.

However, the concentration of these pH buffering agents is still not enough for electroporation applications because a large amount of H^+^ and OH^−^ are generated dramatically and locally. Thus H_2_PO_4_^−^ and HPO_4_^2−^ ions will be depleted in the area near to the electrodes. Compared with the Eppendorf hypoosmolar electroporation buffer, the modified buffer (the detailed composition is listed in Table 1) contains more KH_2_PO_4_ and K_2_HPO_4_ to contribute a better capacity of pH buffering. Besides a better pH buffering capacity, the modified electroporation also excludes KCl. Thus there is no Cl_2_ or HClO generated at the anodes. This is another advantage of the modified buffer, since HClO is also harmful to cells due to its strong oxidizing property. The other component myo-inositol is used to adjust the osmolarity.

**Figure 6:**
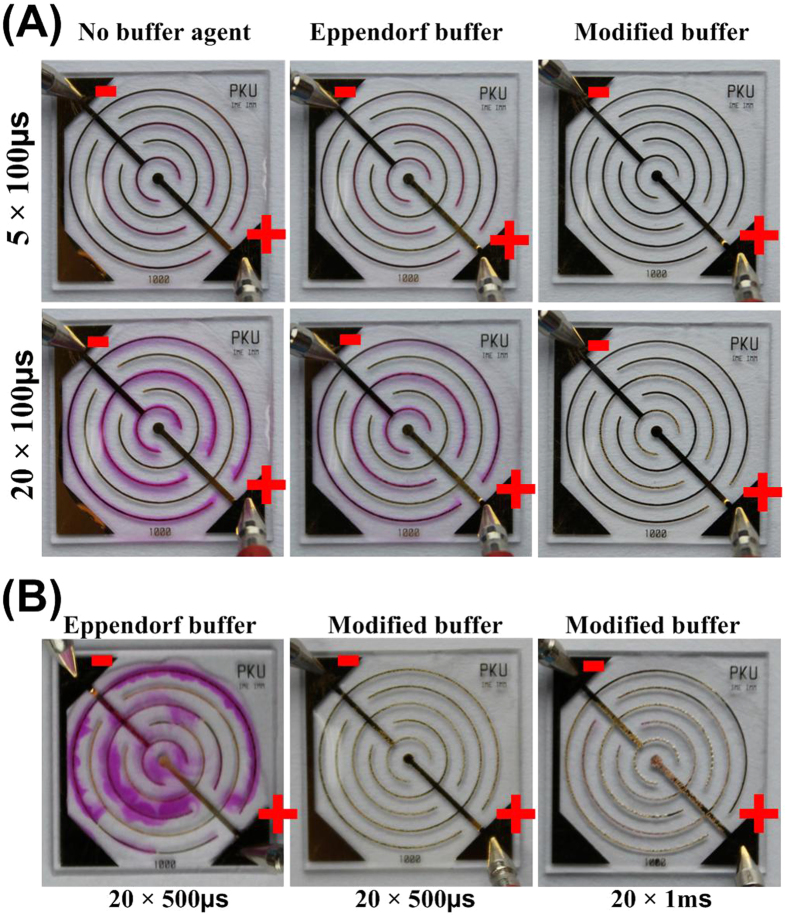
The capacity of pH buffering. (**A**) 100 μs pulse is applied 5 times and 20 times to the microchips, no color change is observed in modified buffer. (**B**) 500 μs and 1 ms pulse is applied 20 times. Bubbles are generated dramatically, whereas the color still not changes in modified buffer.

The pH buffering capacity of modified buffer is tested by using annular interdigitated microchip and pH indicator. The KCl solution without phosphate buffered saline and Eppendorf hypoosmolar buffer are used as controls. The results are shown in Fig. 6(A,B). When electric pulse is applied, localized color change is observed in both KCl solution and Eppendorf buffer, whereas no changes in the modified buffer. Then the duration of electric pulse is increased to 500 μs or 1 ms. A lot of bubbles come out, known as hydrogen and oxygen, and attach on the electrodes, because the amount of hydrogen and oxygen exceed the limits that can be dissolved in water. However, the pH value still maintains at a range lower than 8.2 since no color change is observed. The detailed images are shown in Supplementary Figure 5.

**Figure 7:**
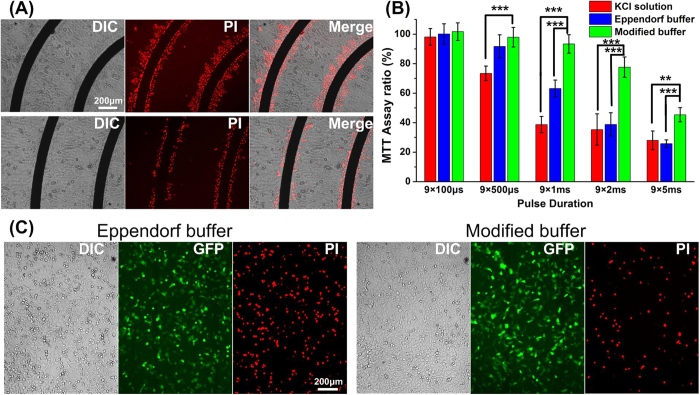
The modified buffer is able to preserve cells from the damage of pH changes during electroporation. (**A**) PI staining images of Hela cells after electroporation. (**B**) Cell viability of electroporation in KCl solution, Eppendorf hypoosomlar buffer and modified buffer. All data is obtained from at least three experiments. **p < 0.05, ***p < 0.01. (**C**) Performance of DNA plasmid electroporation of HEK-293A cells using Eppendorf buffer and modified buffer. The conditions are 100 V, 100 μs, 6 pulses.

We then tested cell viability of electroporation using the modified buffer. Annular interdigitated microchip and high-density distributed electrode network (HDEN)^45^ are used. The PI image indicates that the distribution of cell death in modified buffer is much less than that without buffer agents (Fig. 7(A)). For HDEN device, the result of MTT assay is shown in Fig. 7(B). For short pulse (100 μs) treatment, there is no remarkable difference among these three kinds of electroporation solutions. However, in longer pulse duration treatment, the modified buffer can still help to maintain high cell viability. Cell viability decreases dramatically in the other two solutions. When the 1 ms electric pulse is applied for 12 times, the cell viability of modified buffer is higher than 90%, while only 60% cell can survive in Eppendorf buffer and less 40% cell survive in the solution without pH buffering agents. Even when the duration of pulse is as long as 2 ms and 5  ms, the modified buffer can still preserve cell viability as nearly 80% and 45%. Thus for those types of cells required long duration electric stimulation, the modified buffer is of obvious advantage to preserve cell viability.

We also tested the electroporation efficiency of modified electroporation buffer by transferring DNA plasmid into HEK293A cells using HDEN devices. The results are shown in Fig. 7(C). Generally, the modified buffer can get the same electroporation efficiency, and at the meantime reduce cell death.

## Conclusions

Cell viability is one of the most important parameters to evaluate the efficiency of electroporation especially for precious cells. In this study, we figured out the mechanism of cell death during electroporation, analyzed the scaling down effect of microchips and proposed a strategy to improve cell viability.

The pH near cathodes and anodes changes dramatically because of electrolysis. The localized strong acidic or alkaline environment is fatal to cells. Severe cell death in alkaline environment is caused by saponification of membrane. The major composition of cell membrane, phosphatidylcholine, decomposed in basic environment, resulting to cell lysis. The pH changes play a dominant role that contributes to cell death during electroporation. On the other hand, the electric filed assists toxicity of pH changes. Electric field makes the membrane bilayer structure more permeable and looser, which is more vulnerable in alkaline environment.

Although the voltage required for electroporation decreases, the scaling down of the microchips contributes to severe side effects. Smaller size of electrodes causes less electric field homogeneity and more segments with pH changes. Thus the distribution of cell death expands. This has a negative effect on transfection efficiency and cell viability.

As we have already known the pH value change is detrimental to cells when electroporation, we make some modifications on the electroporation solution used previously. We exclude KCl and increase the content of KH_2_PO_4_ and K_2_HPO_4_ to improve the capacity of pH buffering. The modified buffer helps to improve cell viability of electroporation. This improvement provides us a chance to improve cell transfection efficiency. As cell viability can be kept in an acceptable level even when the applied electroporation parameters are rigorous, such as several microsecond long pulse duration and more than 10 pulse number.

## Materials and Methods

### Cell culture

We used HEK-293A and HeLa cells in our experiment. Both of them were originally purchased from the Chinese Academy of Medical Science and then are cultured by ourselves in DMEM medium supplemented with 10% fetal bovine serum, 100 units/mL penicillin, and 100 mg/mL streptomycin (Life Technologies, Gibco). All the results are valid for HEK-293A and HeLa cells in this manuscript.

### Fabrication of Microchips

The fabrication process of microchips is the standard micromachining technique. A glass wafer is used as substrate. Then a layer of chrome and gold is sputtered on the substrate. Chrome is served as adhesive layer. And gold is used for its bio-compatibility. The thickness of chrome and gold layer is 30 nm and 300 nm respectively. Then the electrodes are defined by photolithography. Gold and chrome layers are patterned by wet etching.

### Demonstration of pH change during electroporation

Phenolphthalein (below pH 8.2: colorless, above pH 10: fuchsia), Congo red (below pH 3.0: blue, above pH 5.2: red), Alizarine yellow R (below pH 10.1: yellow, above pH 12: red) are used as pH indicators. We performed electroporation on the angular formatted interdigitated microchips with solution contained pH indicator and recorded the color change on the microchips during the pulse number course. Eppendorf hypoosomlar electroporation buffer (Eppendorf, Hamburg, Germany) is used as medium. The electric conditions are 120 V and 100 μs, which means the electric field is about 1.0 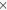 10^5^ V/m. The electric field intensity is equivalent to the optimum condition proposed by our previous studies. Electric stimulation is performed by ECM-830 stimulator (BTX, USA).

### Determination of the effect of pH changes on cell viability

To evaluate the cytotoxicity of acid and base, cells are plated in 96-well plates at 1 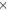 10^4^ cells per 100 μl DMEM per well one day before MTT assay. HEK-293A and Hela cells are treated with solution of series of pH value (1, 1.5, 2, 2.5, 3, 4, 7.4, 10, 10.5, 11, 11.5, 12, 12.5, 13) for different time including 10 seconds, 30  seconds and 2 minutes. The solution with different pH is prepared by KCl and KOH. And the pH value is verified by pH meter. After this treatment, each well is washed by 200 μl DMEM twice. For MTT assay, 200 μl fresh DMEM medium is added and cells are continued to culture overnight. Then, 200 μl medium is removed and MTT (100 μl fresh medium contained 2 μl MTT) is added into each well. Plates are incubated for 4 hours, after which all medium is removed and 50 μl DMSO is added into each well and further incubated for 30 minutes. Finally, the absorbance is read at 540 nm with a reference wavelength of 650 nm and the absolute absorbance (ODnet540) is OD540 minus OD650. For comparison of relative viability, all data are presented as the mean percentage ± SEM in pentaplicate samples compared to the absorbance value of mock-treated cells. MTT ratio which indicates cell viability is calculated as:


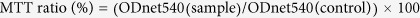


Where ODnet540(sample) is the absorbance at 540 nm of the treated cells and ODnet540(control) is the absorbance at 540 nm of the mock control (non-treated cells).

Besides, we also seed HEK-293A cells on the annular interdigitated microchip and treated cells with solution of series of pH value (12, 12.5, 13) for 10 second. Then cells are washed with DMEM twice. After that, cell morphology is observed under microscope. PI staining is added five minutes before observation.

### Determination of cell death distribution after electroporation on microchips

Cells were cultured on the microchip and observed *in-situ* to manifest the localized distribution of cell death. HeLa cells are harvested and resuspended at a density of 1 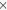 10^5^ cells per 1 ml. Each microchip is fixed on the bottom of each well of 12-well plates. Then, 200 μl mixture is added on each microchip and cells are incubated for 4 hours for cells to adhere, after which 1 ml DMEM is added per well and incubated overnight. Before being applied with electric pulses, cells are washed with electroporation buffer for twice and 20 μl electroporation buffer is dropped on a chip. Electric stimulation is applied by ECM-830 stimulator (BTX, USA). The conditions are 60 V, 100  μs, and three pulses. Immediately after electroporation, 1 mL of cell culture medium is added into each well and cells are incubated for another 5 min, to allow the recovery of the temporarily increased permeability of cell membrane. Then 1 ml medium is removed and another 1 ml PI containing fresh medium is added into each wells. PI fluorescence is recorded by a fluorescence microscopy (Olympus IX-71).

## Additional Information

**How to cite this article**: Li, Y. *et al.* Electroporation on microchips: the harmful effects of pH changes and scaling down. *Sci. Rep.* **5**, 17817; doi: 10.1038/srep17817 (2015).

## Supplementary Material

Supplementary Information

## Figures and Tables

**Table 1:** Composition of electroporation buffers.

	KCl	KH_2_PO_4_	K_2_HPO_4_	myo-Inositol	pH value	Osmolarity (mOsmol/kg)
Solution without phosphate saline	27 mM	0	0	36 mM	7.0	~90
Eppendorf hypoosmolar electroporation buffer*	25 mM	0.3 mM	0.85 mM	36 mM	7.4	~90
Modified electroporation buffer	0	13 mM	13 mM	25 mM	7.2	~90

^*^From “Ependorf Multiporator Basic Applications Manual”, page 49.
