# Supplementary material for: Electroporation on microchips: the harmful effects of pH changes and scaling down
Source: Sci Rep. 2015 Dec 14;5:17817. doi: 10.1038/srep17817 (PMC4677314; doi:10.1038/srep17817)
Supplement: Supplementary Information [file srep17817-s1.doc]

Electroporation on microchips: the harmful effects of pH changes and scaling down

*Yang Li,^a†^ Mengxi Wu^b,c†^ Deyao Zhao,^a^ Zewen Wei,^d^ Wenfeng Zhong,^b^  Xiaoxia Wang,^a^ Zicai Liang^a*^ and Zhihong Li^b*^*

^a^Institute of Molecular Medicine, Peking University, Beijing 100871, China

^b^National Key Laboratory of Science and Technology on Micro/Nano Fabrication, Institute of Microelectronics, Peking University, Beijing 100871, China

^c^Department of Engineering Science and Mechanics, The Pennsylvania State University, State College, PA 16801, USA

^d^National Center for Nanoscience and Technology, Beijing 100190, China

^†^These authors contribute equally to this work.

**SUPPLEMENTARY INFORMATION**

**SUPPLEMENTARY FIGURES**


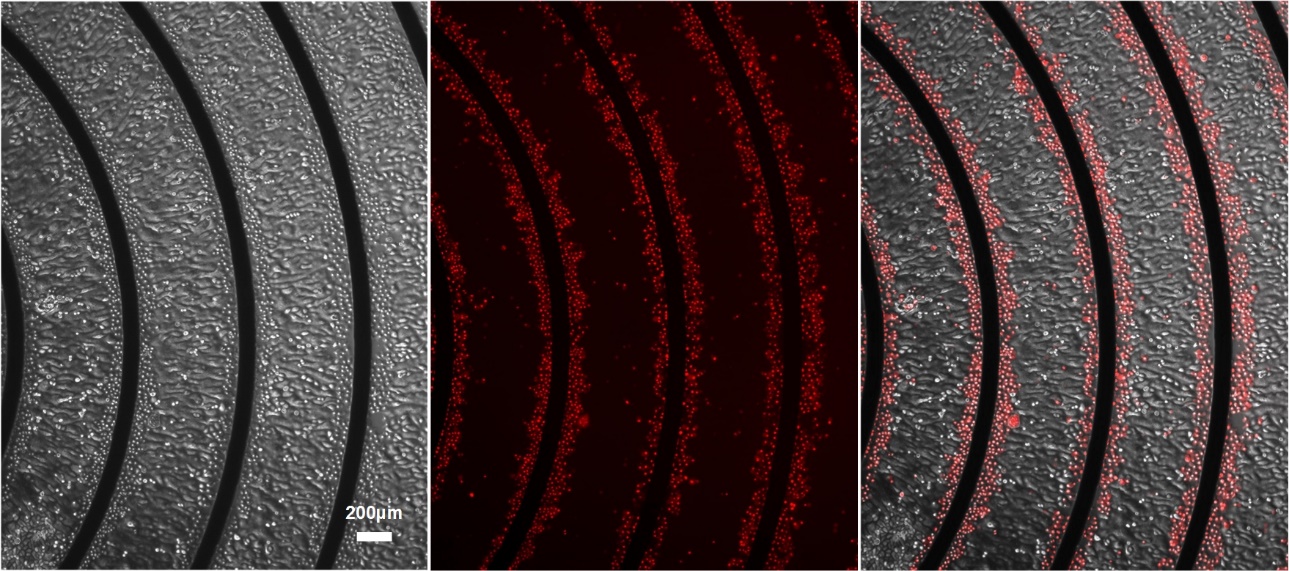


***Supplementary Figure 1.*** The localized death distribution of Hela cells 1h after electroporation. The gap between electrodes is 500μm. The electric conditions are 60V, 100μs, 9 pulses. PI is added to indicate the dead cells. In terms of morphology, cells near both anodes and cathodes shrink. The distribution of dead cells, which is stained by PI, occupies about 100μm of the annular area near the electrodes.

***
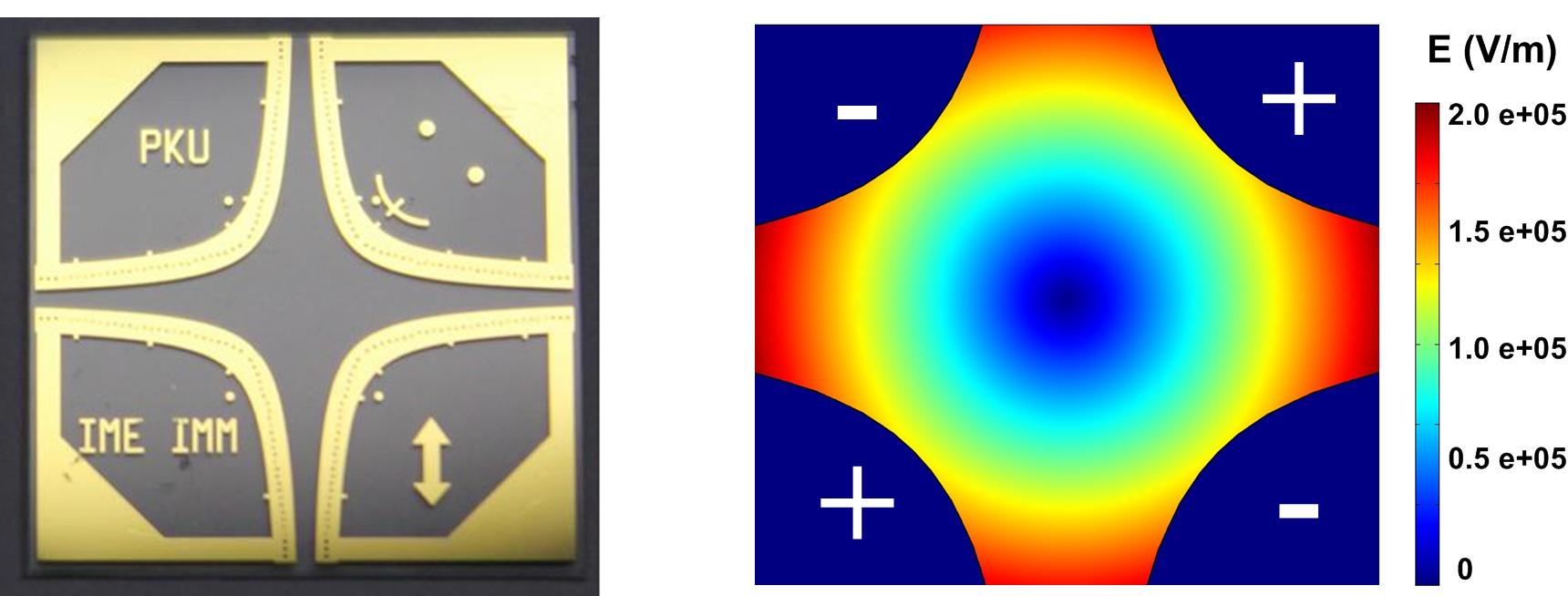
***

***Supplementary Figure 2.*** Electric field intensity distribution of four-leaf microchip. Four hyperbolic formatted electrodes are patterned symmetrically. Annular gradient electric field distribution is generated by applying voltage between adjacent electrodes.

***
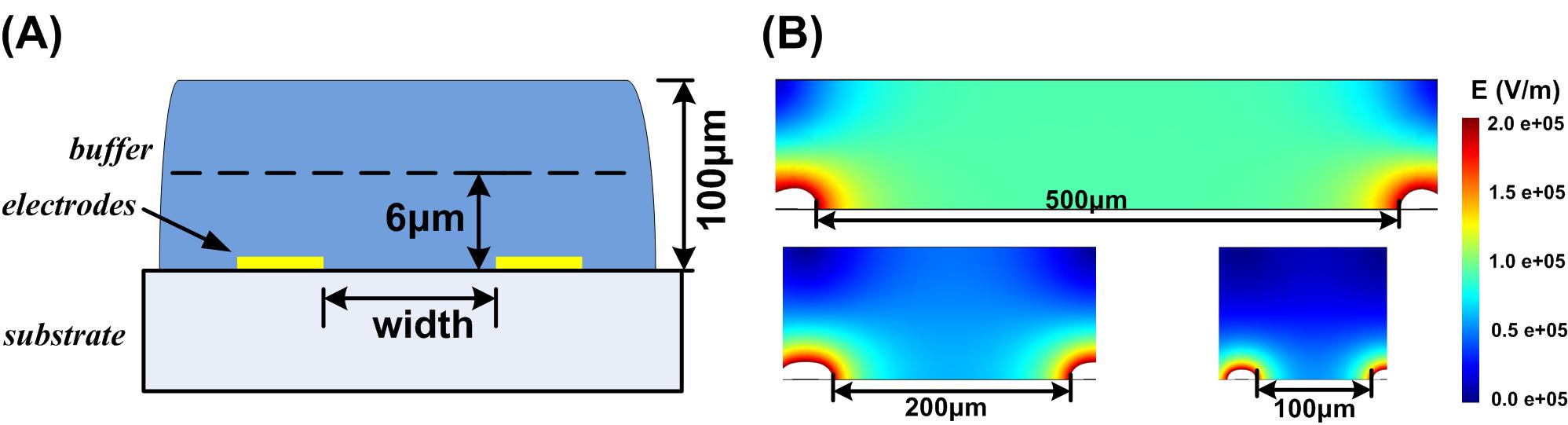
***

***Supplementary Figure 3.*** Distribution of electric field on microchips. (A) Schematic of the electric model for micromachined electroporation chips. (B) The cross section view of electric field distribution. The width between two electrodes is 500μm, 200μm and 100μm respectively. More uniform electric field is generated by 500μm electrodes.


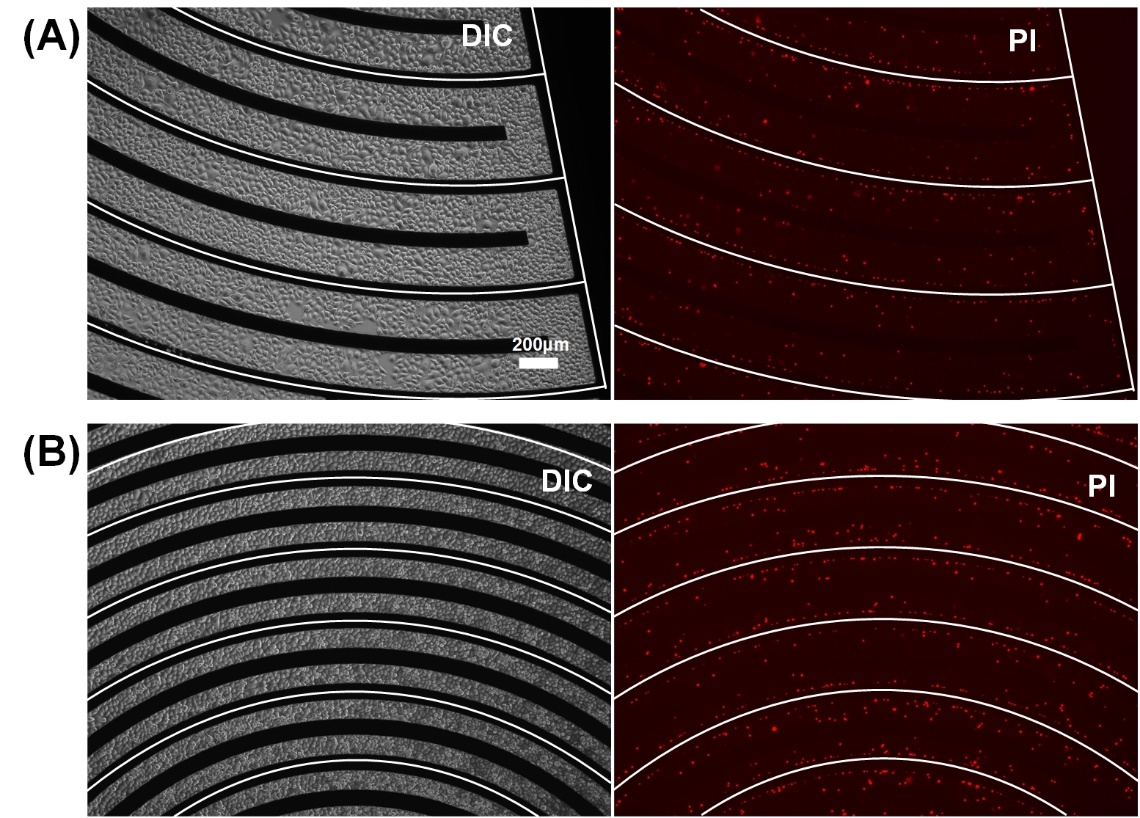


***Supplementary Figure 4.*** Images of Hela cells 24h after electroporation. The gap between electrodes is 200μm (A) and 100μm (B). The electric conditions are 36V, 100μs for 200μm microchips and 20V, 100μs for 100μm microchips. The electrodes indicated by white line are cathodes. The dead cells are mainly distributed near the cathodes.


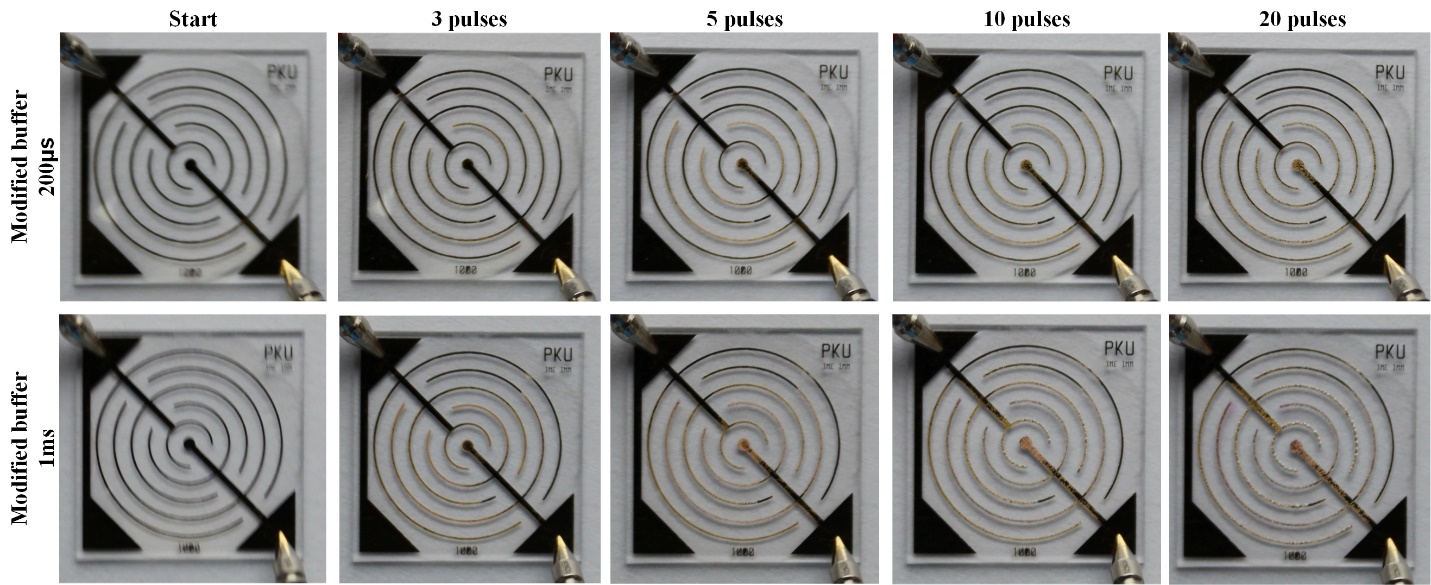


***Supplementary Figure 5.*** The modified buffer has a better pH buffering capacity. The gap of electrodes is 1000μm. The electric conditions are 120V, 200μs and 120V, 1ms respectively. Though a large number of bubbles are generated, no color change is observed after 20 pulses.
